# Dual targeting of solid tumors using cytokine-induced killer cells modified with a CAR anti-tenascin C and a secretable EGFRxCD3 bispecific antibody

**DOI:** 10.1007/s00262-025-04149-2

**Published:** 2025-09-13

**Authors:** Silvia Zaninelli, Irene Cattaneo, Rut Valgardsdottir, Roberta Frapolli, Ezia Bello, Marina Meroni, Andrea Gianatti, Silvia Panna, Paola Rizzo, Susanna Tomasoni, Maurizio D’Incalci, Martino Introna, Josée Golay, Alessandro Rambaldi

**Affiliations:** 1 Center of Cellular Therapy “G. Lanzani”, Department of Oncology and Hematology, ASST Papa Giovanni XXIII, Bergamo, Italy; 2 University of Milano-Bicocca, Milan, Italy; 3 Laboratory of Cancer Pharmacology, Department of Oncology, Istituto di Ricerche Farmacologiche Mario Negri IRCCS, Milan, Italy; 4 Pathology Unit, ASST Papa Giovanni XXIII, Bergamo, Italy; 5 Istituto di Ricerche Farmacologiche Mario Negri IRCCS, 24100 Bergamo, Italy; 6 Department of Biomedical Sciences, Humanitas University, Milan, Italy; 7 IRCCS Humanitas Research Hospital, Rozzano, Milan, Italy; 8 Department of Oncology and Hematology, ASST Papa Giovanni XXIII, Bergamo, Italy; 9 Department of Oncology and Hematology, Università Degli Studi Di Milano, Milan, Italy

**Keywords:** Chimeric antigen receptor, Tenascin C, Cytokine induced killer, Bispecific T cell engager, solid tumor

## Abstract

To generate CARCIK cells with enhanced targeting and penetration of solid tumors, we have designed new CAR molecules against tenascin C (TNC), an extracellular matrix and surface molecule, overexpressed in several tumor types. Two different anti-TNC CAR constructs were generated, sharing the anti-TNC scFv domain, fused to the CD3ζ fragment, but differing in co-signaling domains: CAR-TNC4 carrying the 4-1BB and CAR-TNC5 the CD28 and OX40 domains. Both CARs were introduced into cytokine-induced killer cells (CIK) by co-transfection with the sleeping beauty transposase plasmid. CARCIK-TNC cells were cytotoxic against TNC^+^ targets, proliferated and secreted the IFN-γ and IL-2 cytokines in response to target cell binding, with overall higher efficacy of CARCIK-TNC5 compared to CARCIK-TNC4. To enhance activity and specificity, we also generated a dual construct, carrying a secretable EGFRxCD3 bispecific T cell engager (sBiTE) antibody cDNA. CIK transfected with EGFRxCD3/CAR-TNC5 showed good expression of both molecules and synergistic killing of MDA-MB-231 cells in vitro compared to cells transfected with the single-targeting molecules. Also in vivo, in the subcutaneous TNC^+^ EGFR^+^ MDA-MB-231 xenograft model, significantly enhanced control of tumor growth was observed after injection of CIK cells transfected with dual, compared to single constructs. We conclude that CAR-TNC5 combined with EGFRxCD3 can endow CIK cells with different and potentially synergistic functions in vivo.

**Supplementary Information:**

The online version contains supplementary material available at 10.1007/s00262-025-04149-2.

## Introduction

Chimeric antigen receptor T cell (CAR-T) technology has revolutionized leukemia immunotherapy [1, 2]. However, many hurdles remain in applying this therapy for solid tumors, in part due to poor infiltration of immune cells [3, 4].

The tumor microenvironment (TME) includes multiple cellular components as well as a non-cellular complex extracellular matrix (ECM), both of which may promote tumor growth and metastasis [5–8]. The ECM has an impact on the immune response, acting both as a barrier that can physically block the immune response and an immunomodulator [9–11]. Tenascin C (TNC) is an attractive ECM molecule for targeted delivery of therapeutics, being overexpressed in several tumor types (glioblastoma, epithelial carcinoma, lymphomas of B and T cell origin, etc.) and associated with tumor progression and metastasis [12, 13]. Several antibodies have been generated against different domains of TNC and shown to efficiently localize to tumor tissue [14, 15]. Among these, ST2146 (tenatumomab) is directed against the EGF-like repeats domain of TNC, present in both large and small isoforms of TNC and has reached phase I study for hepatocarcinoma treatment as radio-conjugate [16, 17]. Several other radio- or cytokine-conjugates of mAbs directed against different TNC domains have been developed, demonstrating good tumor localization compared to normal tissue [12].

Cytokine-induced killer (CIK) cells are a subtype of cytotoxic CD3^+^CD56^+^ T cells expanded in vitro after IFN-ɣ stimulation and capable of infiltrating both hematopoietic and solid tumors and inducing anti-tumor activity without significant graft-versus-host disease (GvHD) [18–24]. In our laboratory, CIK cells modified with an anti-CD19 CAR molecule have been generated using electroporation of a Sleeping Beauty transposon system and used successfully to treat relapsed and refractory B-cell acute lymphoblastic leukemia (B-ALL) patients in an allogeneic setting (Eudract n. 2017-000900-38 and 2020-005025-85) [25–28].

Given the positive results obtained with CAR-T CD19 and CARCIK-CD19 in clinical trials, we set out to extend this technology to new CAR molecules against TNC, using the tenatumomab sequence to generate the anti-TNC single-chain fragment variable (scFv). We analyzed the functionality of 2 CARs carrying the anti-TNC scFv upstream of different signaling modules and CAR backbones, inserted into CIK cells through a transposon system. The 2 CARs were based on 2 CAR-CD19 molecules that have been used successfully in the clinic, tisagenlecleucel and the CAR-CD19 developed by our groups.[28, 29] Finally, we have generated a dual targeting construct to enhance the activity of the best CAR-TNC, by adding a secreted bispecific T-cell engager (sBiTE) to the transfected plasmid. These were tested against solid tumor cell lines in vitro and in vivo in a xenograft model.

## Materials and methods

### Cells

The human colon adenocarcinoma HT29 and T lymphoblastic CEM cell lines were cultured in RPMI 1640 medium (Euroclone, Wetherby, West Yorkshire, UK) supplemented with 10% heat-inactivated fetal bovine serum (FBS) (Euroclone), 2 mM L-glutamine (Euroclone) and 100 μM gentamycin (PHT Pharma, Milano, Italy). The human breast cancer MDA-MB-231 and the embryonic kidney HEK-293 cell lines were maintained in α-MEM medium (Gibco, Thermo Fisher Scientific, Waltham, Massachusetts, USA) supplemented with 10% heat-inactivated FBS (Euroclone) and 100 μM gentamycin (PHT Pharma). Peripheral blood mononuclear cells (PBMCs) were purified by Ficoll-Hypaque (Lympholyte-H; Cedarlane, Burlington, Canada) gradient centrifugation of normal donors’ buffy coats, obtained after informed consent.

### Detection of TNC

To detect extracellular or intracellular TNC by flow cytometry, either live or fixed and permeabilized cells (Cytofix/Cytoperm kit, BD Bioscience, San José, CA) were stained with the murine anti-human TNC Mab (BC-24) (Invitrogen), followed by the FITC-labeled goat anti-mouse IgG.

For in situ immunofluorescent staining, cells were seeded onto sterile cover slips in 6-well plates in complete medium for 1–4 days. Cells were then fixed with 4% paraformaldehyde (Thermo Fisher) and blocked with PBS + 5% FBS. Cells were incubated with the mouse anti-human TNC mAb (BC-24), followed by the Cy3-labeled donkey anti-mouse secondary antibody (Jackson ImmunoResearch, Ely, Cambridgeshire, UK) and finally with 4’,6-diamidino-2-phenylindole dihydrochloride (DAPI, Merck KGaA, Darmstadt, Germany). The coverslips were finally mounted on slides and observed on an inverted fluorescence microscope (Axio Vert A1, Zeiss, Oberkochen, Germany) and a confocal microscope (confocal laser scanning SP8, Leica, Wetzlar, Germany).

To quantify TNC expression, reverse transcription quantitative polymerase chain reaction (RT-qPCR) was performed on the RNA extracted from different cell lines with the RNeasy mini kit (QIAGEN, Hilden, Germany). RNA was retrotranscribed using the SuperScript IV VILO Master Mix (Thermo Fisher), following the manufacturer’s instructions. Amplifications were carried out in Power SYBR green PCR Master Mix (Applied Biosystems, Thermo Fisher) with TNC-specific primers (Suppl. Figure S3) or primers for 2 reference genes, the β-glucuronidase gene (GUS) and the human glyceraldehyde 3-phosphate dehydrogenase gene (GAPDH). The relative expression levels of TNC among different cell lines were analyzed using the 2^−ΔΔCt^ method.

### Generation of cell lines stably expressing TNC

To generate target cells stably expressing the TNC antigen, an expression construct carrying the EGFR-like repeats domain of TNC fused to the trans-membrane and intracellular region of the heparin-binding epidermal growth factor (HB-EGF-TM) was synthesized (GeneArt, Thermo Fisher). The HB-EGF intracellular region was mutated to avoid the nuclear signaling, as already reported in literature [30]. The fused cDNA was cloned into the pT4 transposable vector carrying the recognition sequences of Sleeping Beauty transposase, to give the pT4-TNC-TM plasmid. CEM and HEK-293 cells were transfected with pT4-TNC-TM and SB100X plasmids, using the nucleofector kit V and Amaxa IIb nucleofector device (Lonza, Basel, Switzerland). After 7–10 days expansion, stably transfected TNC-TM^+^ cells were purified by immunoselection with magnetic beads (Miltenyi Biotec). CEM and HEK-293 cell lines stably expressing the TNC-TM fusion protein on > 90% of cells were obtained.

### Generation of anti-TNC transposon plasmids and CARCIK cells

**Fig. 1:**
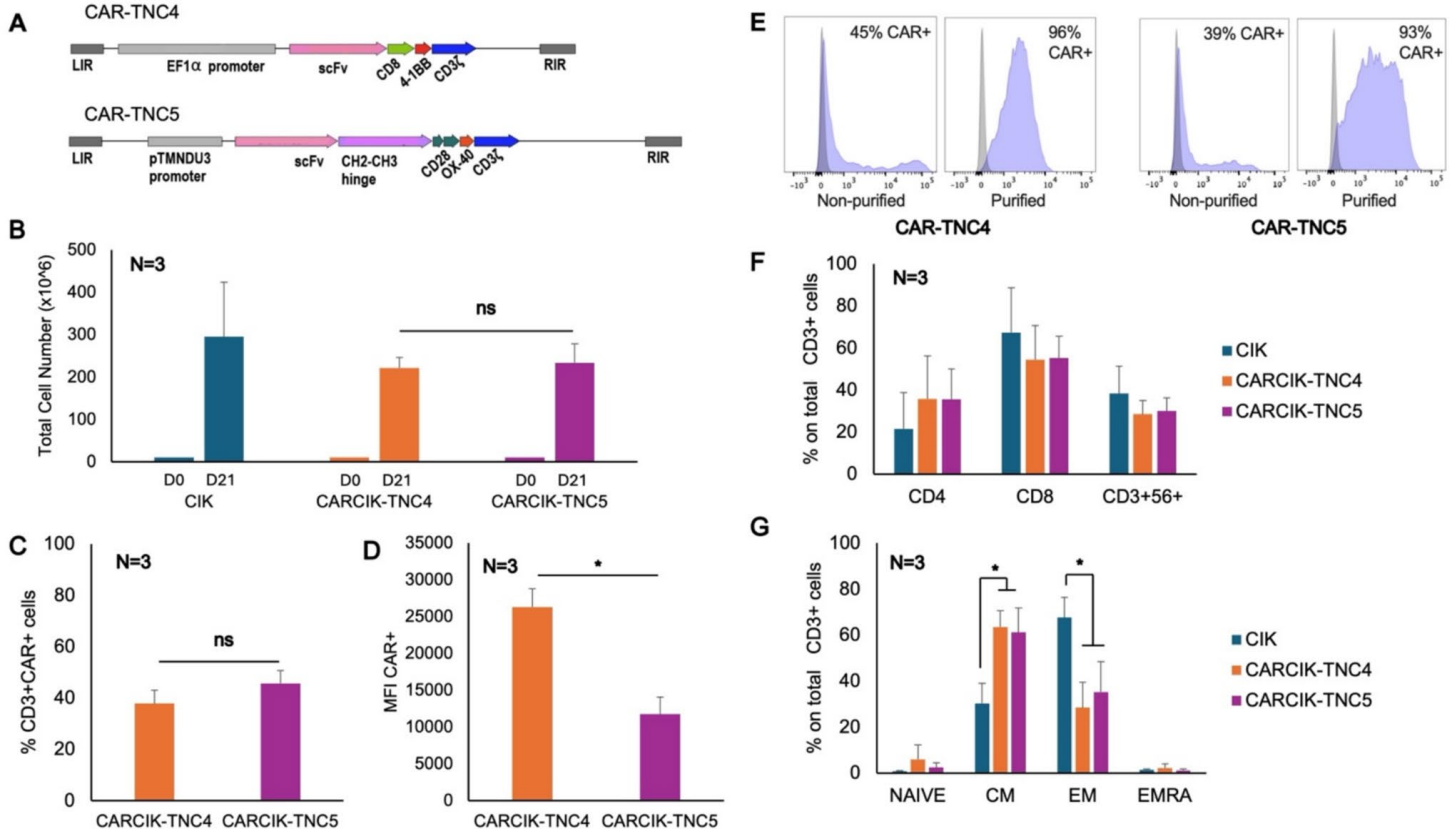
Generation of CARCIK-TNC cells and their characterization. **A** Schematic structure of the CAR-TNC constructs. **B** 10 × 10^6^ PBMCs were co-transfected with the CAR-TNC4 and CAR-TNC5 + SB100X plasmids and expanded to CIK for 21 days. Data report total nucleated cells obtained. **C**-**D** CAR expression was analyzed at the end of culture by flow cytometry. Percentage CAR positive **C** and mean fluorescence intensity of CAR **D** are shown. **E** Examples of flow cytometry histograms of purified or non-purified CARCIK-TNC4 (MFI 29803 and 2086) or -5 (MFI 8928 and 4054). **F**-**G** Immunophenotype of the purified cellular products at the end of the culture, including CD4, CD8 and CD3^+^CD56^+^ populations **F** and effector-memory populations **G**, analyzed by flow cytometry. The results are the means and standard deviations of 3 experiments using different donors as starting material. The statistics refer to the comparison between CARCIK-TNC4 and CARCIK-TNC5 with unmodified CIK (*p < 0.05)

We generated two different anti-TNC CAR constructs, expressing the anti-TNC single-chain fragment variable (scFv) based on the VH and VL tenanumomab sequence, and fused to 2 different spacer, trans-membrane and intracellular signaling domains (see sequences in Fig. 1A and Supplementary Figure S3). The SB100X transposase plasmid pCMV(CAT)T7-SB100 was a gift from Zsuzsanna Izsvak (Addgene plasmid #34,879; http://n2t.net/addgene:34879; RRID:Addgene_34879) [31].

CARCIK-TNC cells were generated as previously described [27]. Briefly, 10 × 10^6^ PBMCs were co-transfected on day 0 or 2 with the transposon vectors carrying the CAR-TNC and/or EGFRxCD3 coding sequences and the SB100X plasmid, using the human T-cell nucleofector kit (Lonza) and the Amaxa IIb nucleofector device (Lonza). Cells were cultured in RPMI 1640 advanced medium (Gibco, Thermo Fisher Scientific) supplemented with 20% FBS (Euroclone) and 2 mM L-Glutamine (Euroclone). 1000 U/mL IFN-γ and 50 ng/mL anti-CD3 (OKT-3, TakaraBio, Kyoto, Japan) were added on dais 1 and 2, respectively. 300 U/ml recombinant human interleukin 2 (rhIL-2, Proleukin, Clinigen Healthcare Ltd) was added 3 times a week. In some cases, 10 days after transfection, anti-TNC CAR^+^ cells were immunoselected using poly-histidine-tagged recombinant human TNC protein (rhTNC-His, R&D Systems), then anti-histidine FITC antibody, anti-FITC magnetic beads and column (Miltenyi Biotec). The positive fraction was collected and maintained in culture until day 21 in a G-Rex device (Wilson Wolf Manufacturing, St Paul, MN). As a control, unmodified CIK cells were expanded in parallel from the same donors.

### CARCIK-TNC immunophenotyping

CARCIK-TNC cells were characterized using the following mAbs: anti-CD3-PerCP-Cy5.5 (SK7 clone), anti-CD56-BV510 (NCAM16.2 clone), anti-CD4-PE-Cy7 (SK3 clone), anti-CD8-APC-H7 (SK1 clone), anti-CD45RA-FITC (L48 clone), anti-CD62L-APC (SK11 clone) (all from BD biosciences). CAR was detected by staining with rhTNC-His and FITC, APC or PE-conjugated anti-histidine antibody (Miltenyi Biotec). EGFR expression was detected with cetuximab and the anti-human IgG-FITC (BD Biosciences). The His-tagged EGFRxCD3 sBiTE was detected in the cell culture supernatant by incubating it with EGFR^+^ cells, followed by APC-conjugated anti-histidine antibody. A FACScanto II flow cytometer device (BD Biosciences) was used to analyze the samples with BD FACSDiva Software.

### In vitro functionality assays

CARCIK-TNC cells were tested, at the end of the culture, for the cytotoxic activity, the proliferation capacity and the cytokine release as already previously described [32–34] and detailed in the Supplementary materials and methods.

### TNC^+^ tumor models in vivo

Experiments in vivo were authorized by the local ethical committee on animal experimentation and the Italian Ministry of Health. Animals were handled following European (EEC Council Directive 2010/63/UE) and Italian (D.lgs 26/2014) laws on animal experimentation. Briefly, NOD-SCID mice (6 weeks-old females, NOD.CB17-*Prkdc*^scid^/NCrHsd, Envigo) were inoculated subcutaneously with 5 × 10^6^ MDA-MB-231 cells. When tumors reached about 250 mg, mice were randomized in groups of 8 mice and treated i.v. either with PBS, 10 × 10^6^ unmodified CIK or 10 × 10^6^ gene-modified non-purified CIK. Cell treatment, generated from the same donor, was repeated three times at 10 days intervals. Tumor growth was measured at least twice a week, and animals euthanized when tumors reached > 1500 mg.

Formalin-fixed tumor specimens were stained with tenatumomab [17] or antibodies against human CD3, human nuclear antigen (HNA) or human granzyme B. The procedure is detailed in the Supplementary materials and methods.

### Statistical analyses

Data were analyzed using the Student’s *t*-test or the unpaired Mann–Whitney test. For in vivo assays, a longitudinal analysis was performed to study the outcome trend over time for different treatments. Linear mixed models were used to test the interaction between time and treatment groups, considering time and group as fixed effects, and subject-specific effects as random effects. All the analyses were performed using R software (version 4.3.0). Growth curves were analyzed using t-tests with Satterthwaite’s method. A significance level of 0.05 was fixed.

## Results

### TNC expression in tumor cell lines

To select tumor cell lines for functional assays, we screened 5 solid tumor cell lines for TNC expression. TNC protein was measured by immunofluorescence, either by flow cytometry of permeabilized cells in suspension, or by staining cells grown on slides for several days and subsequently fixed. TNC protein was detected at variable intensity in 4/5 solid tumor cells lines: HT29, Hep-G2, HT-1080 and MDA-MB-231 (Table 1). The confocal microscopy images for HT29 and MDA-MB-231 are shown in supplementary Figure S1. A weak and strong TNC RNA expression, respectively, was detected by RT-qPCR in HT29 and MDA-MB231 (Table 1).

**Table 1:** TNC expression in tumor cell lines

Tumor cell line	Tumor subtype^a^	Flow Cytometry (permeabilized)^b^	Immunofluorescence on slides (formalin-fixed)	qPCR^c^
HT29	CRC	ND	+	+
Hep-G2	Hepatocarcinoma	ND	+	ND
MDA-MB-231	BC	ND	+	+ +
MCF7	BC	−	ND	ND
HT-1080	Fibrosarcoma	±	ND	ND

^a^BC: Breast carcinoma; CRC: colorectal carcinoma

^b^Flow cytometry: + + + : > 69%; + + : 30–59%; + : 20–29%; ± : 5–19%; −: < 5%. ND: Not done

^c^qPCR: + + : > 10; + : 1–10; ± : 0.1–0.9; −: < 0.1 (HT29 was used as the control cell line)

ND: Not done

### Generation of stably transfected cell lines expressing TNC-TM fusion protein

To generate target cells stably expressing surface TNC as model systems in functional assays, the CEM and HEK-293 cell lines were stably transfected with a plasmid carrying the cDNA for the EGF-like repeats domain of TNC, recognized by tenatumomab, fused to the HB-EGF trans-membrane domain and called pTNC-TM. Cells were immunoselected to > 90% purity and expressed the TNC domain on their cell surface, as shown by immunostaining (Supplementary Figure S2).

### Generation and characterization of CARCIK-TNC cells

We generated a novel anti-TNC CAR based on the V_H_ and V_L_ sequences of anti-TNC mAb tenatumomab. The anti-TNC scFv was then inserted upstream of two different CAR backbones described previously [35], and shown schematically in Fig. 1A. The first construct, CAR-TNC4, contains the CD8 hinge and trans-membrane domains and the 4-1BB and CD3ζ signaling domains based on the clinically approved anti-CD19 CAR produced by Novartis (Tisagenlecleucel) [36]. The second construct, CAR-TNC5, carries the human IgG1 hinge and CH2-CH3 domains as spacer element, followed by the CD28 trans-membrane and signaling domains and finally the OX40 and CD3ζ signaling domains and recapitulates the anti-CD19 CAR used in the CARCIK-CD19 clinical trial [27]. The sequences are provided in Supplementary Figure S3 (Fig. 1).

We next generated CIK cells genetically modified by electroporation of the 2 different anti-TNC CARs and SB100X transposase plasmids and culture for 21 days in CIK conditions. Both CARCIK-TNC4 and CARCIK-TNC5 cells showed efficient expansion, reaching after 21 days a mean 221 × 10^6^ and 233 × 10^6^ total nucleated cells, respectively, starting from 10 × 10^6^ PBMC (Fig. 1B). The expression levels of CAR-TNC4 and CAR-TNC5 were stable and after 21 days reached 34.5% and 43.8% (Fig. 1C), with a mean fluorescent intensities (MFI) of 24,655 and 11,657, respectively (Fig. 1D). CAR-TNC4 is expressed at a slightly lower percentage, but with a significantly higher fluorescent intensity than CAR-TNC5 (*p* < 0.05).

Considering the variability of CAR expression between experiments and between the two different CARs, we decided to routinely purify CAR^+^ cells half-way during culture in order to perform functional assays in vitro on CARCIK cells expressing comparable amounts of CARs. Adding the purification step yielded products expressing > 90% of CAR-TNC in all cases with similar MFI (Fig. 1E and data not shown).

We then characterized the purified CARCIK cell products. The CD4^+^, CD8^+^ and CD3^+^CD56^+^ subpopulations were comparable between both CARCIK-TNC and unmodified CIK cells (Fig. 1F). Regarding effector-memory phenotype, CIK, CARCIK-TNC4 and CARCIK-TNC5 cells were similar for naïve and EMRA subpopulations. In contrast, the central memory (CM) population was significantly more represented for both CARCIK-TNC, being a mean of 63.5% for CARCIK-TNC4 and 61.2% for CARCIK-TNC5, compared to unmodified CIK cells (30.3%) (*p* < 0.05). The percentage of effector-memory cells (EM) was less represented in both CARCIK-TNC, compared to unmodified CIK cells (respectively 28.4%, 35.2% compared to 67.6%, *p* < 0.05, Fig. 1G).

### Cytotoxic activity of CARCIK-TNC cells

**Fig. 2:**
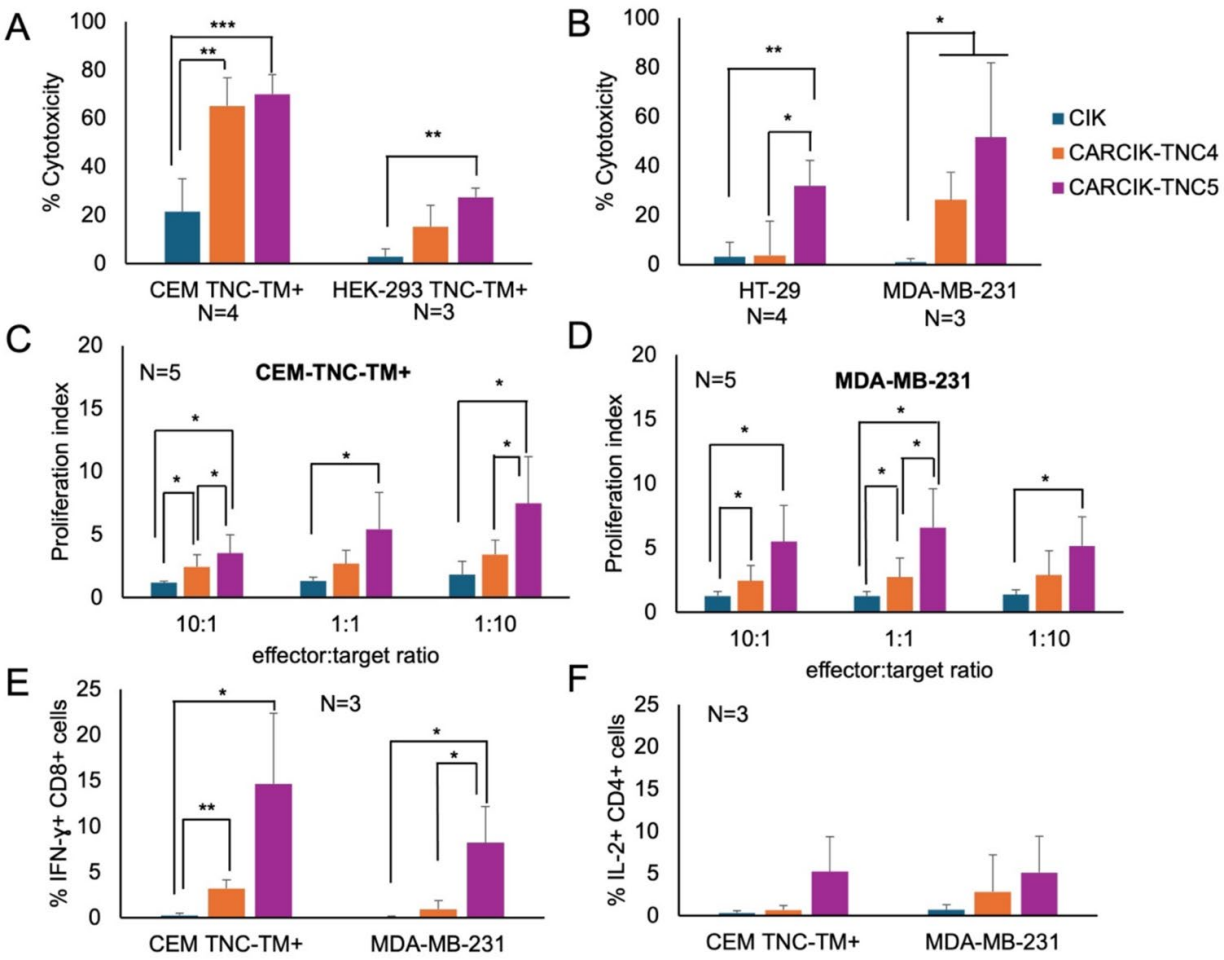
Functional activity of unmodified or CAR-modified CIK cells. **A**-**B** Cytotoxicity of unmodified or CAR-modified CIK in vitro was measured against two TNC stably transfected cell lines, at 5:1 E:T ratio for 4 h **A** or against TNC^+^ tumor cells lines at 1:1 E:T ratio for 24 h **B**. **C**, **D** Proliferation of CAR-modified, CFSE-stained CIK cells in vitro was measured by flow cytometry after stimulation with CEM TNC-TM^+^ **C** or MDA-MB-231 **D**. **E**, **F** IFN-γ **E** and IL-2 **F** production was determined by intracytoplasmic staining upon 6 h co-culture at 1:1 E:T ratio of unmodified or CAR-modified CIK cells. (*P < 0.05, **P < 0.01, ***P < 0.001)

CARCIK-TNC4 and CARCIK-TNC5 cells were evaluated in short (4 h) and long-term assays (24 h) for their cytotoxic effect in vitro against either two TNC-TM^+^ stably transfected cell lines or tumor cell lines naturally expressing TNC. Both CARCIK-TNC cells were strongly cytotoxic against CEM-TNC-TM^+^, compared to unmodified CIK cells (*p* < 0.01, Fig. 2A). Cytotoxic activity against the HEK-293 TNC-TM^+^ target was lower, with only CARCIK-TNC5 cells showing significant killing activity compared to unmodified CIK cells (*p* < 0.05, Fig. 2A). CARCIK-TNC5 cells were significantly more cytotoxic than unmodified CIK cells against both HT29 and MDA-MB-231 cell lines (*p* < 0.01 and *p* < 0.05, respectively, Fig. 2B). In contrast, CARCIK-TNC4 cells were effective only against MDA-MB-231, which expresses higher levels of TNC (*p* < 0.05, Fig. 2B and Table 1).

### Proliferation of CARCIK-TNC cells in response to TNC^+^ target

The proliferative response of CARCIK-TNC cells to the TNC antigen was evaluated using CFSE-labeled CARCIK-TNC cells and either CEM-TNC-TM + (Fig. 2C) or MDA-MB-231 cells (Fig. 2D) as targets. In both cases, CARCIK-TNC5 showed a higher proliferation index compared to unmodified CIK cells or CARCIK-TNC4 cells, although the difference with the latter was not always significant (Fig. 2C and D). Proliferation was in all cases higher at low E:T ratio (1:10) than at the higher E:T ratio (10:1), indicating that CARCIK-TNC5 cells proliferated significantly more in the presence of higher amount of antigen.

### IFN-γ and IL-2 release by CARCIK-TNC in response to TNC^+^ target cells

The cytokines released by CARCIK-TNC4 and -TNC5 cells in the presence of target antigen was evaluated. In the presence of either CEM TNC-TM^+^, both CARCIK-TNC4 and -TNC5 CD8^+^ cells produced more IFNγ than unmodified CIK cells (*p* < 0.05, Fig. 2E). IFN-ɣ was induced also in presence of MDA-MB-231, albeit to lower levels (Fig. 2E).

The release of IL-2 was evaluated on CD4^+^ cells. There was a tendency for CARCIK-TNC5 to induce more IL-2 than CIK or CARCIK-TNC4 cells, but statistical significance was not reached (Fig. 2F).

To summarize, CARCIK-TNC5 cells were overall more effective in vitro than CARCIK-TNC4 cells in terms of cytotoxicity, proliferation, and cytokine release. Nonetheless, CARCIK-TNC4 cells were more effective than non-genetically modified CIK cells in most of these functions.

### Armored CIK genetically modified by dual CAR/EGFRxCD3 constructs

Preliminary experiments in vivo showed that CARCIK-TNC5 cells could reach and infiltrate the tumor (data not shown) but were not sufficient to control tumor growth in long term (Figure S4). Importantly, the cells were non-toxic for the animals, with no major decrease in body weight, even though CAR-TNC should recognize murine TNC like its parent tenatumomab antibody [13].

**Fig. 3:**
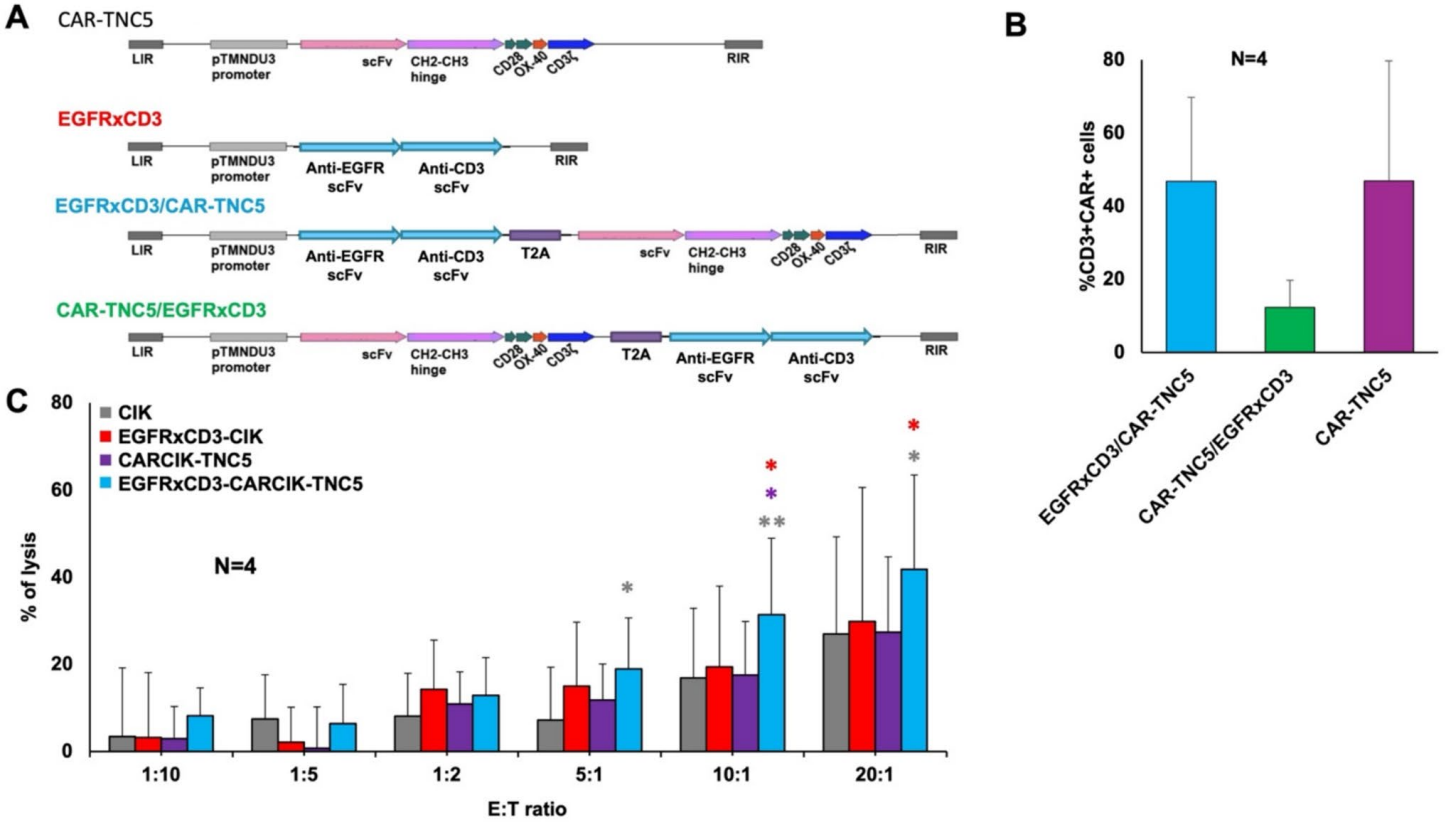
Generation and characterization of armored CIK cells carrying anti-TNC CAR and secretable EGFRxCD3 bispecific antibody. **A** Schematic representation of the different constructs used. **B** TNC CAR expression on CIK cells genetically modified with the different constructs. **C** In vitro cytotoxic activity of unmodified or CAR/EGFRxCD3 modified CIK against the MDA-MB-231 cell line after 4 h of co-culture (N = 4). *: *p* ≤ 0.05 and **: *p* < 0.01. Statistical significance shown refers to armored CIK (i.e., EGFRxCD3/CARCIK-TNC5) against either unmodified CIK (gray *), EGFRxCD3-CIK (red *) or CARCIK-TNC5 (violet *)

To enhance the anti-tumor activity of CARCIK-TNC cells in vivo, we developed a novel armored CAR strategy. The cDNA of a secretable EGFRxCD3 BiTE was placed in the vector either alone, or upstream or downstream of the CAR-TNC5 cDNA, separated by a furin-T2A sequence (Fig. 3A). After transfection of the plasmids into PBMCs and differentiation to CIK, the sBiTE could in all cases be detected in the culture supernatant (Supplementary Figure S5). In the dual constructs, the CAR-TNC5 molecule was found to be better expressed on the surface when the EGFRxCD3 sBiTE sequence was placed upstream, rather than downstream of CAR-TNC5 (Fig. 3B). The EGFRxCD3/CAR-TNC5 construct was therefore selected for further experiments. The CD4/CD8 ratio and naïve-memory phenotypes were not significantly different between CARCIK-TNC5 and armored EGFRxCD3-CARCIK-TNC cells (data not shown).

We then tested the cytotoxic activity of modified CIK in vitro using the MDA-MB-231 cell line, which is positive for both TNC and EGFR (Suppl. Fig. S6 and S1) and 4 h calcein release assays at different E:T ratios. CIK cells modified with either soluble EGFRxCD3 or CAR-TNC5 alone induced only limited target cell death in this short-term killing assay (Fig. 3C). In contrast, dual armored CIK cells showed significant killing compared to CIK modified with the single constructs (*p* < 0.05 at 10:1 E:T ratio). Similar results were obtained at lower E:T ratios in long-term (48 h) killing assays and flow cytometry analysis (data not shown). These data suggest a synergistic effect of combined EGFRxCD3 sBiTE and CAR-TNC5 in tumor cell killing in vitro.

**Fig. 4:**
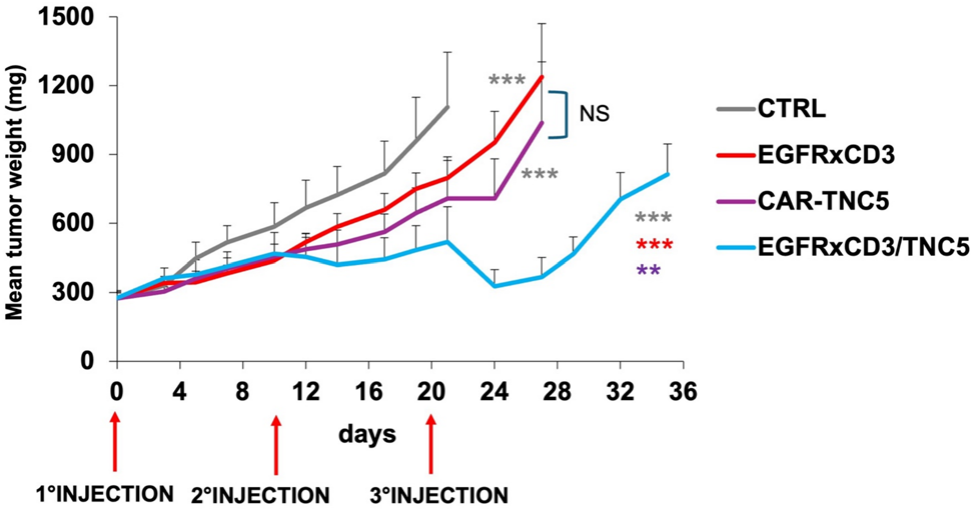
In vivo therapeutic activity of armored CIK or CIK modified with a single-targeting molecule. NOD-SCID mice were inoculated subcutaneously with MDA-MB-231 tumor cells. When tumor reached ~ 250 mg, mice were randomized and inoculated iv with 10 × 10^6^ gene-modified effector CARCIK cells, 3 times at 10 days intervals, as indicated in the graph. Tumor growth curves are shown. Statistical significance refers to armored CIK (i.e. EGFRxCD3-CARCIK-TNC5) against either EGFRxCD3-CIK (red *) or CARCIK-TNC5 (violet *). Significance was also measured against controls group (gray *). **: *p* < 0.01 and ***p < 0.001

Armored CIK cells were then also tested in vivo, in a subcutaneous MDA-MB-231 model in NOD-SCID mice. When tumor volume reached about 250 mg, animals were randomized (day 0) and then inoculated with 10 × 10^6^ CIK cells modified with the single or dual armored EGFRxCD3/CAR-TNC5 constructs. The same treatment was repeated at day 10 and day 20. Whereas CIK expressing CAR-TNC5 or secreted EGFRxCD3 could partially control tumor expansion (*p* < 0.0001 and *p* = 0.0006 respectively versus control group), the dual armored strategy was significantly more effective (Fig. 4, *p* = 0.002 versus CAR-TNC5, *p* < 0.0001 versus sBiTE or control group).

**Fig. 5:**
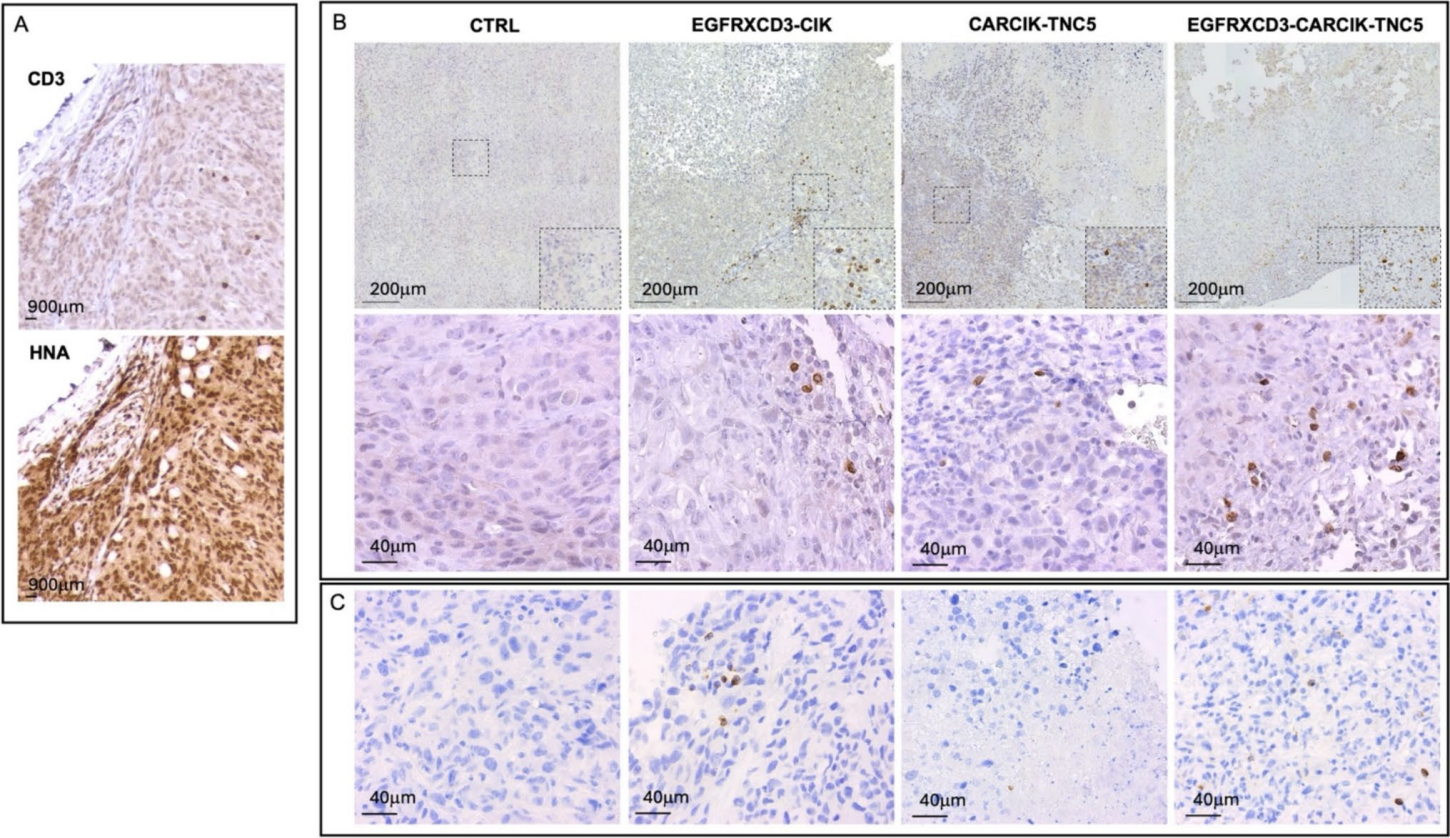
Immunohistochemistry analysis of CIK cells infiltrating the MDA-MB-231 tumor in vivo 24 h after the last effector cells injection. **A** Analysis of human CD3^+^ CIK (upper panel) and HNA^+^ tumor cells (lower panel) on consecutive sections of tumor tissue (original magnification 10x). **B** Representative images of tumor tissue areas characterized by the presence of human CD3^+^ CIK cells. Top panels: mosaic reconstruction (original magnification 20x). Bottom panels: high magnification of representative tumor tissue portions (original magnification 40x). **C** High magnification of representative portions of tumor tissue reveals the presence of secretory granules containing human granzymes B. (original magnification 40x)

We finally verified that CAR-TNC cells inoculated i.v. could infiltrate the tumor tissue in vivo*.* For this purpose, we sacrificed some treated animals either 24 h or 21 days (2–3 animals/group) after the last effector cell injection. Immunohistochemistry analysis of human CD3^+^ cells and total human cells (HNA^+^) was performed in 2 consecutive tumor sections. CD3^+^ gene-modified CIK cells could be detected in the HNA^+^ tumor tissue at both time points, showing that the effector cells infiltrated the tumor and remained there for a long period. Quantification of CD3^+^ infiltration showed no statistically significant difference in the extent of infiltration of CIK cells modified with the different constructs (Fig. 5 and Table 2). Moreover, human granzyme B staining of tumor tissue revealed positive cells in all the treated groups, confirming the human T cells activation in situ, in response to the antigen recognition (Fig. 5C).

**Table 2:** Immunohistochemistry analysis of CIK cell infiltrating MDA-MB-231 tumor in vivo

Inoculated CIK cells	Mean count of CD3^+^ cells/ field (sd)	
	24 h	21 days
EGFRxCD3-CIK	1.8 (0.8)	0.8 (0.5)
CARCIK-TNC5	0.3 (0.5)	0.2 (0.2)
EGFRxCD3/CARCIK-TNC5	1.3 (0.7)	0.5 (0.3)

We conclude that CIK cells genetically modified with the dual EGFRxCD3/CAR-TNC5 construct infiltrated the tumor and were very effective in controlling tumor growth, whereas single constructs showed only partial activity. These data suggest a synergistic effect of EGFRxCD3 and CAR-TNC in the armored CARCIK strategy for the control of TNC^+^ tumors in vivo.

## Discussion

The use of CAR-T cells has achieved impressive clinical results in some patients with otherwise incurable lymphoid malignancies. Unfortunately, very few data are available for solid tumors, which usually remain resistant to cellular immunotherapy due to the lack of tumor-specific antigen targets and the difficulty for immune cells to penetrate and be activated in the solid tumor masses. To favor the tumor infiltration by cytotoxic effector T cells, we generated CARs targeting TNC, an extracellular matrix protein abundantly expressed in the tumor microenvironment of both hematologic and solid cancers [37–39]. We chose CIK cells as the cytotoxic T effector cells to be genetically modified with the novel CAR-TNC, using a non-viral, transposase-based system [25, 26].

Two anti-TNC CARs were designed and successfully expressed by CIK cells, the CAR expression leading to a shift in the effector/memory phenotypes, with diminished EM and increased CM subsets, compared to untransduced CIK cells. These differences are probably due to the basal signaling produced by CARs, even in the absence of target cells. An increased CM population has been reported to induce a longer persistence in vivo [40].

Purified CARCIK-TNC cells were active in vitro in terms of cytotoxicity, proliferation and cytokine release in response to stably transfected or native TNC^+^ cell lines. CARCIK-TNC5 consistently demonstrated higher overall efficacy over CARCIK-TNC4, despite showing > 90% CAR expression in both cases and similar MFI. The higher functional activity of CAR-TNC5 could be due to either the different hinge or costimulatory domains. The hinge, derived from the CD8 molecule for TNC4 and CH2-CH3 for TNC5, can induce a different flexibility of the constructs, thereby improving the construct affinity for the target antigen [41]. At the same time, the different costimulatory domains can play an important role in driving effector functions. In particular, the combination of CD28 and OX40 in the CAR-TNC5 intracellular domain has been reported to increase the release of IL-2 tenfold compared to the CD28 signal alone [42]. These results are in line with those obtained with similar CAR constructs directed against CD19 [35]. This confirms that, in vitro*,* the response of the construct based on the molecule carrying the CH2-CH3 spacer and CD28-OX40 co-signaling modules performs better than the 41-BB carrying construct. Comparison of the activity of different anti-CD19 CAR-T cells signaling domains have been reported in the literature [43, 44]. In agreement with these studies, CD28 induces a higher release of cytokines compared to 4-1BB and has a higher activity in vitro. In contrast 4-1BB is associated to a better persistence in vivo in patients [45, 46].

With regard to in vivo experiments, CIK modified with CAR-TNC alone were able to infiltrate the neoplastic masses. They were not toxic for the animals, even though the ani-TNC moiety should also recognize the murine TNC. However, their anti-tumor activity was not significant. For this reason, we developed a dual armored construct by adding to the CAR-TNC a secretable, scFv-based, EGFRxCD3 bispecific T cell-engaging antibody (sBiTE).

The construct that most efficiently expressed both molecules was that with the sBiTE placed upstream of the CAR (EGFRxCD3/CAR-TNC5). CIK stably transfected with this construct induced a synergistic cytotoxic activity in vitro against the TNC^+^EGFR^+^ MDA-MB-231 tumor target, compared to either CIK carrying the sBiTE or CAR-TNC5 alone. Furthermore, when tested in the subcutaneous MDA-MB-231 model in NOD-SCID mice, CIK-EGFRxCD3/CAR-TNC5 controlled tumor cell growth significantly more efficiently than CIK cells genetically modified with either EGFRxCD3 or CAR-TNC5 alone. Analysis of human CD3^+^ cells in the tumor tissues demonstrated that armored EGFRxCD3/CARCIK-TNC5 cells do indeed infiltrate the tumors and remain detectable up to 21 days after inoculation. Furthermore, cytotoxic activity of the infiltrated CIK cells could be demonstrated in situ, as shown by human granzyme B staining.

Altogether, these data suggest that armored EGFRxCD3/CARCIK-TNC5 cells have a synergistic action both in vitro and in vivo, thanks to the separate and different action of both targeting molecules. On one side, the CAR-TNC may favor tumor localization, direct anti-tumor cytotoxicity, and pro-inflammatory cytokine release upon target recognition. Antigen binding also induces CARCIK-TNC proliferation. On the other side, the co-expressed, secretable EGFRxCD3 moiety may induce further killing of EGFR^+^ tumor cells in situ, mediated by both the gene-modified CIK cells themselves, but also by non-modified CIK cells, since these will be activated by the anti-CD3 moiety of the sBiTE, as already reported in similar settings [47]. In a fully human context, the EGFRxCD3 would also allow endogenous cytotoxic T cells within the tumor to become activated and cytotoxic against tumor cells. The use of an immunodeficient mouse model allowed us a first evaluation of the activity of our CARCIK-TNC cells in combination with the sBiTE in vivo, but a humanized patient-derived xenograft (PDX) model will be necessary to confirm the synergistic activity of the dual construct also in presence of endogenous T cells. Such model is complex to set up and beyond the scope of this report.

Recently, a CAR targeting the C domain of TNC has been developed and retrovirally transduced into T cells. This construct showed limited efficacy in a xenograft tumor model in vivo, similar to ours, but was effective when combined with a constitutively active IL-18R [48]. This latter CAR is directed against a different, alternatively spliced domain of TNC. The CAR-TNC construct used here, in contrast, targets the N-terminal EGF-like repeats domain of TNC which is expressed in all TNC isoforms. TNC is overexpressed in tumors compared to normal adult tissues [38, 49]. We did not observe significant toxicity of our CAR-TNC, even though it can recognize both the human and mouse protein, expressed in healthy tissues [50, 51]. Although by itself, our anti-TNC CAR appeared not to be sufficient for effective control of TNC^+^ tumors in vivo, its combination with secretable EGFRxCD3 is shown here to be very promising and is a novel synergistic approach. A similar dual CAR/BiTE has been proposed recently, combining an anti-EGFRvIII CAR with a secretable EGFRxCD3 BiTE in a bicistronic lentiviral vector. The addition of EGFRxCD3 sBiTE was shown to enhance anti-tumor activity of the transfected cells and engage also non gene-modified T cells, in vitro and in vivo.[47] Also, an anti-MUC16 CAR construct combined with a secretable anti-WT1 BiTE has been proposed recently [52]. There are clear differences between these latter proposals and ours: we used CIK cells as effectors, which have by themselves a propensity to localize to tumor tissue [18]; we used a CAR directed against an ECM molecule (TNC) rather than a tumor antigen, which may favor this localization to tumors as well as convey direct anti-tumor activity; we have added a secretable EGFRxCD3 BiTE which adds a different mechanism of cytotoxicity to the gene-modified CIK cells concerning the CAR molecule; finally we used a viral-free transposon system for gene modification. TNC is not only overexpressed in tumor tissue, enhancing the localization of CAR-TNC modified CIK cells to the tumor, but is also involved in tissue remodeling and metastasis [38, 53]. Targeting this molecule may therefore provide the effectors with functions different from direct tumor cell killing.

The lack of a specific mechanism to limit the constitutive secretion of the EGFRxCD3 BiTE outside the tumor mass may be considered as a potential limit. On the other hand, it may represent an effective and long-lasting delivery of a potent antineoplastic drug.

The armored approach described here, of a CAR targeting an ECM protein combined with a secreted BiTE, has been deposited as a patent. This strategy may indeed be envisioned to be extended to other tumor types that are strongly TNC positive, such as glioblastoma, combining the CAR-TNC with sBiTEs targeting different tumor antigens, such as GD2 [53]. Furthermore, the same approach could be applied to other ECM molecules that are overexpressed in tumors.

## Supplementary Information

Below is the link to the electronic supplementary material.

Supplementary file1 (DOCX 1368 KB)

Supplementary file2 (PDF 235 KB)

## Data Availability

The data generated in this study are available upon request from the corresponding author.
