# Supplementary material for: Dual targeting of solid tumors using cytokine-induced killer cells modified with a CAR anti-tenascin C and a secretable EGFRxCD3 bispecific antibody
Source: Cancer Immunol Immunother. 2025 Sep 13;74(10):305. doi: 10.1007/s00262-025-04149-2 (PMC12433391; doi:10.1007/s00262-025-04149-2)
Supplement: Supplementary file 1 — Supplementary file1 (DOCX 1368 KB) [file 262_2025_4149_MOESM1_ESM.docx]

**Cancer Immunology, Immunotherapy (submitted in 2025) - Silvia Zaninelli et al.**

**Supplementary Materials and Methods**

*Cytotoxicity assay*

Cell lysis was evaluated using the GFP-certified^TM^ Apoptosis/Necrosis detection kit (Enzo Life Science, Farmingdale, NY, USA). TNC^+^ target cell lines were first labeled with the green fluorescent dye 5(6)-Carboxyfluorescein diacetate *N-*succinimidyl ester (CFSE; Sigma-Aldrich, Merck KGaA). The CEM-TNC-TM^+^ and HEK-293-TNC-TM^+^ cell lines were stained with 0.5 μM CFSE immediately prior to testing, while the HT-29 and MDA-MB-231 cell lines, naturally expressing TNC, were stained with 1 μM CFSE, plated and allowed to reach confluency for four days in culture. Gene modified or unmodified CIK cells were then co-cultured with CFSE-labeled targets at a 5:1, 1:1, down to 1:10 effector:target (E:T) ratio for 4-24 hours. Cells were then collected, washed and stained with the Apoptosis Detection Reagent (Annexin V-Enzo Gold) and the Necrosis Detection Reagent for 10 minutes and analyzed by flow cytometry. The percentage of dead cells was determined by calculating the overall percentage of Annexin V^+^ and Necrosis Detection Reagent^+^ in CFSE^+^ target cells co-cultured with the effectors minus the spontaneous lysis of target cells alone. Maximal lysis was measured by adding Apoptosis Inducer (staurosporine) in positive control wells.

To detect synergy *in vitro* with combined secreted EGFRxCD3 sBiTE and CAR-TNC, standard calcein-AM cytotoxicity assays were performed. Briefly, MDA-MB-231 cells were plated and allowed to reach confluency. After four days of culture, target cells were labeled with 1 µM Calcein-AM (Fluka, Sigma-Aldrich) and washed twice in complete medium. Labeled cells were then incubated at 37°C, 5% CO2, in the presence of gene-modified or unmodified CIK cells at different effector:target ratios. After 4 h, the cells were sedimented by centrifugation, 100 µL supernatant was collected, and calcein release was determined using a fluorescence microplate reader (Fluostar Optima, BMG Labtech, Ortenberg, Germany) (excitation: 485 nm and emission: 535 nm). The percentage of specific calcein release was calculated as % specific lysis with the formula:

$$\% specific lysis=100x\frac{\left( test release - spontaneous release \right)}{\left( maximal release - spontaneous release \right)}$$

*Proliferation assays*

The proliferation of CARCIK-TNC cells following target recognition was evaluated using CFSE. Briefly, CIK and CARCIK-TNC cells were stained with 1 μM CFSE and plated at 0.4x10^6^ cells/well in presence or absence of target cells. In the case of HT-29 and MDA-MB-231 target cell lines were plated overnight prior to adding CARCIK-TNC to let them adhere to plastic and express TNC extracellularly. After 4 days co-culture, cells were collected and stained with CD3-PE, CD4-PE-Cy7 and CD8-APC-H7 antibodies (BD Bioscience) and CFSE expression was analyzed on different populations by flow cytometry, using the ModFit LT^TM^ software to calculate the proliferation index.

*Cytokine induction*

The ability of CARCIK-TNC cells to produce cytokines in response to target cells was evaluated by intracellular staining and flow cytometry. HT-29 and MDA-MB-231 cells were plated for four days prior to testing to reach confluency and express TNC extracellularly. Effector and target cells were co-cultured for six hours at a 1:1 E:T ratio in presence of BD GolgiStop solution (BD Bioscience). Cells were then collected, fixed and permeabilized using the BD Cytofix/Cytoperm kit (BD Bioscience) following the manufacturer’s instructions and stained with CD3-PerCP-Cy5.5, CD4-PE-Cy7, CD8-APC-H7 (BD Bioscience), IFN-γ-FITC and IL-2-PE antibodies (Miltenyi Biotec). The samples were then analyzed by flow cytometry.

*Immunohistochemistry analysis*

CIK cell infiltration in tumor tissue was analyzed in 3 µm-thick formalin-fixed, paraffin embedded (FFPE) sections, after staining for cells expressing human CD3 (CIK) and Human Nuclear Antigen (HNA)(tumor). After deparaffinization and rehydration in decreasing alcohols, antigen retrieval was performed using a Decloaking chamber (DC NxGen 220V, Biocare Medical) for 15 minutes at 110°C with DIVA decloaking buffer pH 6.0 (Biocare Medical). Endogenous peroxidases were then quenched by incubating sections for 5 minutes with peroxidazed1 (Biocare Medical). After blocking non-specific sites with Background Punisher (Biocare Medical), sections were incubated with LN10 mouse anti-human CD3 (1:100, Biocare Medical) or mouse anti-HNA (Sigma Aldrich) or mouse anti-human Granzyme B (1:100, Leica Biosytems), followed by MACH4-HRP Polymer kit (Biocare Medical) and diaminobenzidine substrate solution. Slides were finally counterstained with Meyer’s hematoxylin, dehydrated in graded alcohols and observed by light microscopy (Apotome Axio Imager Z2, Zeiss). Some tissue portions were imaged as mosaic reconstruction through a motorized stage using an EC Plan-Neofluar 20x objective lens and Axiovision 4.8.2 acquisition software (Zeiss).

CD3^+^ cells were quantified throughout the sections as the number of observed CD3^+^ cells in each high-power field (HPF – 40x objective), by considering, for every sample, two independent sections cut at different depths. The extent of CD3^+^ cells infiltration was expressed as the mean count of CD3^+^ cells per field.

**Supplementary Figures**

**
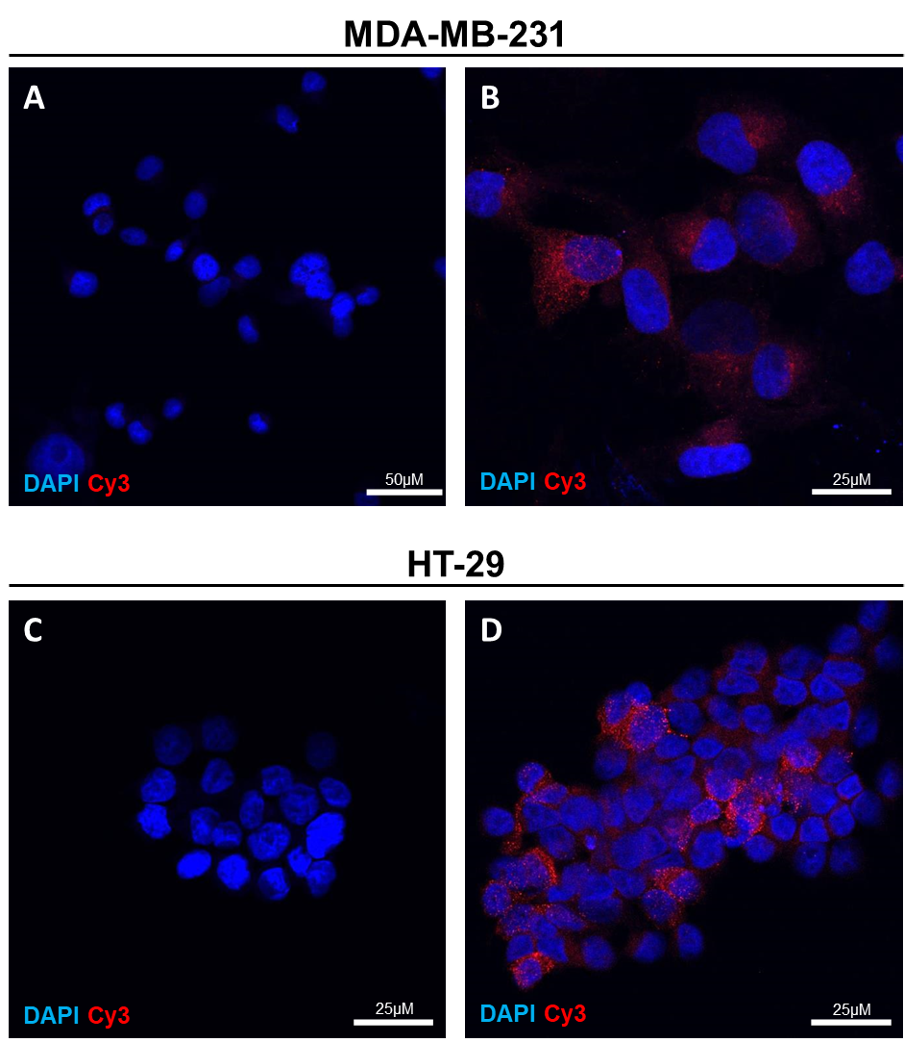
**

**Supplementary figure S1: TNC protein expression in 2 solid tumor cell lines.**

The indicated cell lines were cultured four days to allow extracellular matrix deposition and were then fixed and stained with anti-TNC antibody, Cy3-labelled secondary antibody and DAPI. (A) and (C) negative controls. (B) and (D) Positive TNC fluorescence (red).

**
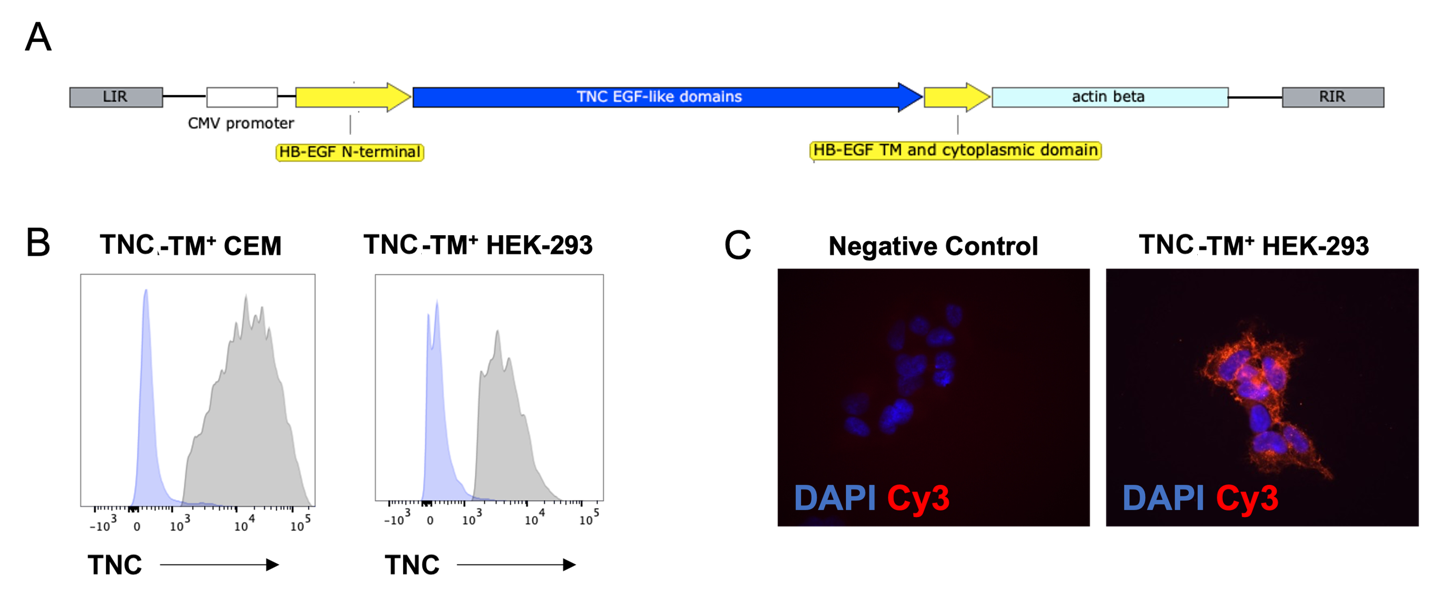
**

**Supplementary figure S2: Generation of two TNC-TM^+^ cell lines.**

(A) Schematic representation of the TNC-TM plasmid construct. (B) TNC-TM expression on stably transfected cell lines was measured by flow cytometry: in grey is anti-TNC detection and in blue the isotype control. (C) Immunofluorescent staining of TNC-TM^+^ HEK-293 cells, Cy3 staining (in red) is for TNC and DAPI (in blue) is for nuclei visualization. Images have been acquired with the inverted fluorescent microscope (63X).

**
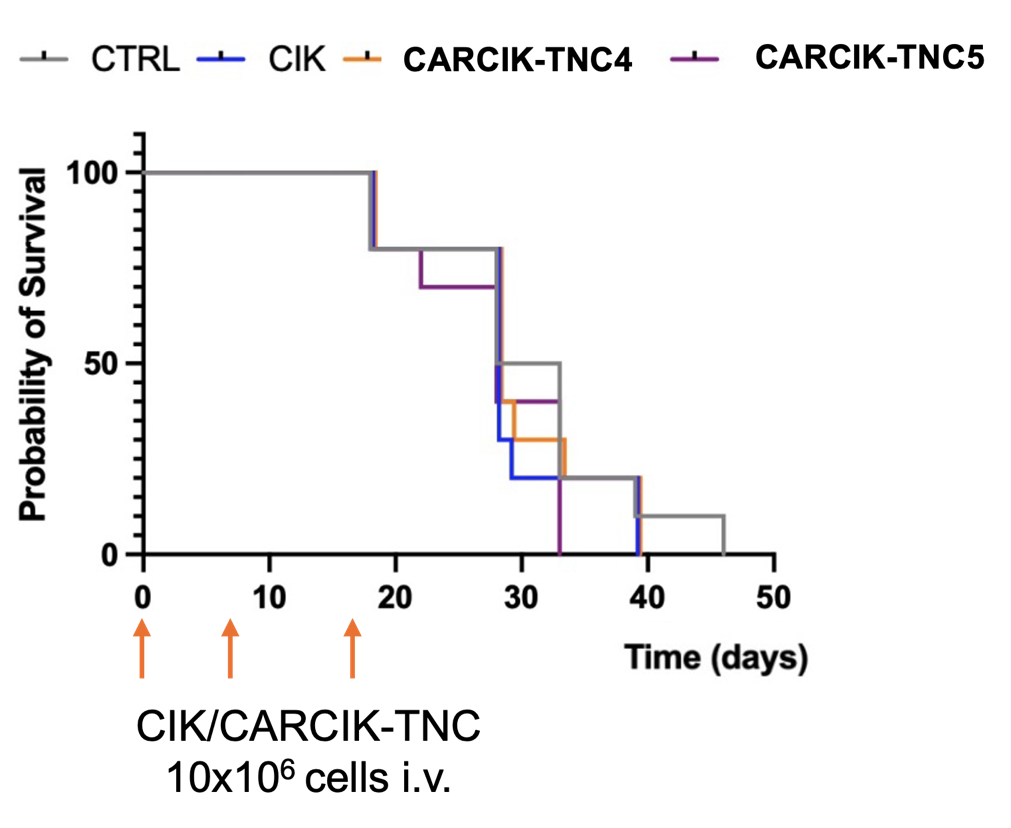
**

**Supplementary Figure S4: *in vivo* preliminary data**

NOD-SCID mice were inoculated with MDA-MB-231 cell lines and treated as shown schematically on the Kaplan-Meier survival curves x axis.

**
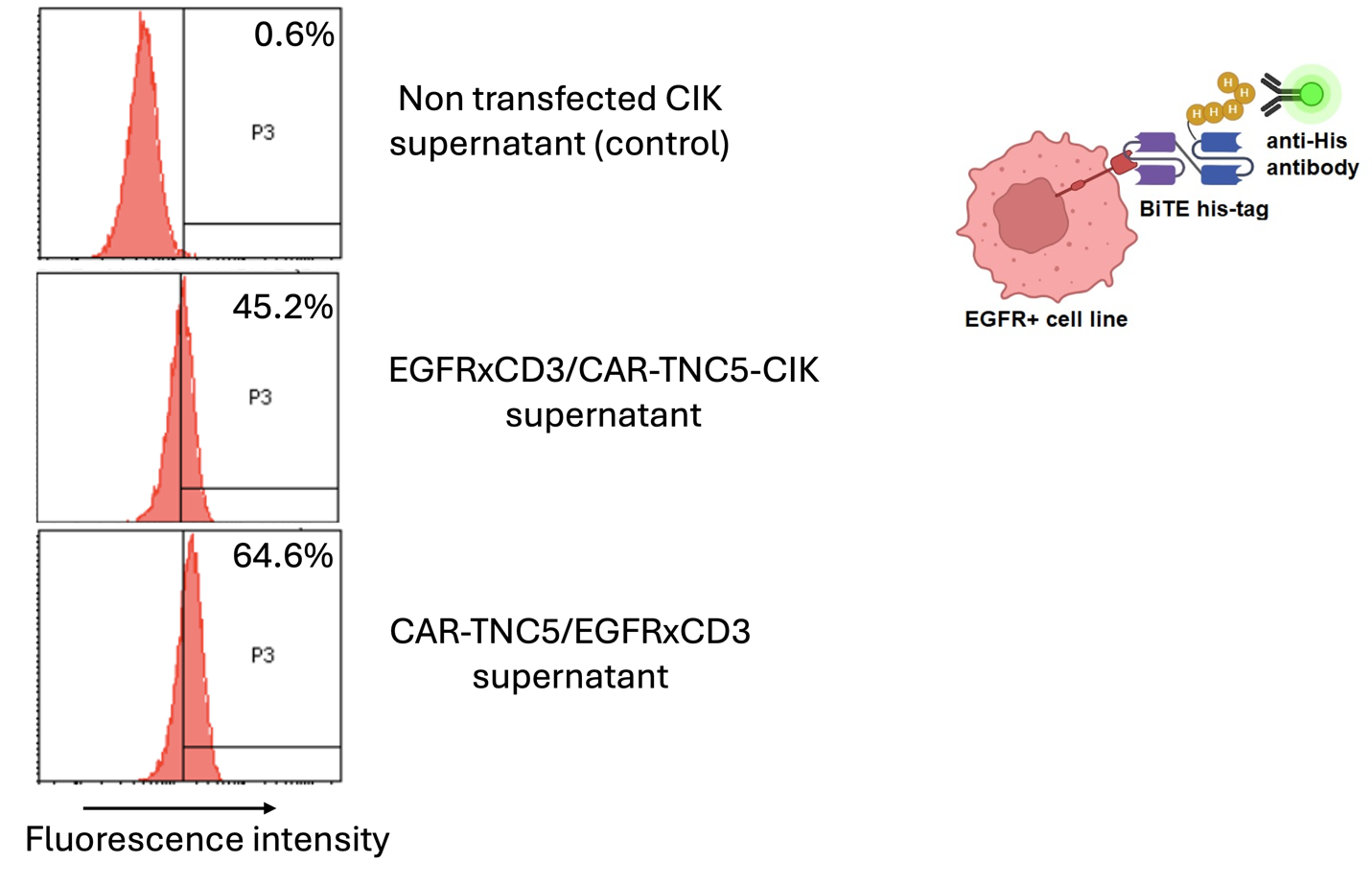
**

**Figure S5. Detection of EGFRxCD3 secreted by transduced CIK cells.**

CIK cells transfected with the EGFRxCD3/CAR-TNC5 or CAR-TNC5/EGFRxCD3 constructs were cultured for 24 hours. Supernatant was then collected and incubated with EGFR+ MDA-MB-231 cells followed by labelled anti-His antibody.

**
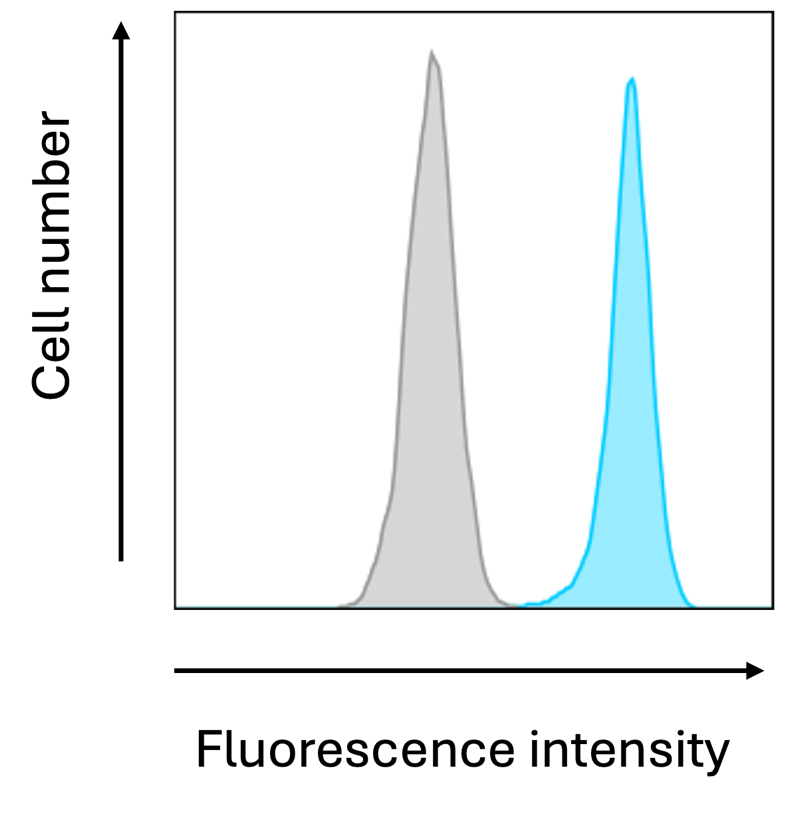
**

**Supplementary Figure S6. EGFR expression in MDA-MB-231 cell line by flow cytometry.**

Blue: MDA-MB-231 cells stained with cetuximab + anti-human IgG-FITC secondary antibody. Grey: negative control, MDA-MB-231 cells stained only with the secondary antibody.
