# Supplementary material for: Dual targeting of solid tumors using cytokine-induced killer cells modified with a CAR anti-tenascin C and a secretable EGFRxCD3 bispecific antibody
Source: Cancer Immunol Immunother. 2025 Sep 13;74(10):305. doi: 10.1007/s00262-025-04149-2 (PMC12433391; doi:10.1007/s00262-025-04149-2)
Supplement: Supplementary file 2 — Supplementary file2 (PDF 235 KB) [file 262_2025_4149_MOESM2_ESM.pdf]

Supplementary Figure S3. Sequences of constructs and primers

1) ***Secretable EGFRxCD3 BiTE coding sequence***

Human Serum Albumin Leader

atgaagtgggtcaccttcatctctctgctcttcctgttttcgagcgcctactcccgcggcgtgttccgccgt

M K W V T F I S L L F L F S S A Y S R G V F R R

Cetuximab VL

gacattctgctgacccagagccccgtgatcctgagcgtttcaccaggtgagcgcgtctccttctcctgccga

D I L L T Q S P V I L S V S P G E R V S F S C R

gcatcccagtccataggcacaaacatccactggtaccagcagcgcaccaatggctccccgcgcctgctcatc

A S Q S I G T N I H W Y Q Q R T N G S P R L L I

aagtacgcgtccgagagcatttccggcatcccctctcgcttcagcgggagtggatctggcaccgacttcact

K Y A S E S I S G I P S R F S G S G S G T D F T

ctgtctattaattctgtggagtctgaagacatcgcggactactattgtcagcagaacaacaactggcctacc

L S I N S V E S E D I A D Y Y C Q Q N N N W P T

Linker (G4S)x3

acgtttggtgccggcactaagctcgagctgaaaggaggcggaggcagcggcgggggcggatctggcggcggt

T F G A G T K L E L K G G G G S G G G G S G G G

Cetuximab-VH

ggttctcaagtgcagttgaagcagtccggacctggactggtgcagccgtcccagagtctttccatcacctgc

G S Q V Q L K Q S G P G L V Q P S Q S L S I T C

accgtgtccggcttctcgctgaccaactacggcgtccactgggtccgccagtcgcccggcaagggcctggag

T V S G F S L T N Y G V H W V R Q S P G K G L E

tggctcggcgtgatttggtctggaggcaacactgattacaacacgcccttcacctcccgcttgagcattaac

W L G V I W S G G N T D Y N T P F T S R L S I N

aaggacaactccaagagccaggtctttttcaagatgaattccttgcagagcaacgacaccgccatctactat

K D N S K S Q V F F K M N S L Q S N D T A I Y Y

tgtgcccgcgccctcacttactacgactacgagttcgcctattggggccagggaacattggtcaccgtgtcc

C A R A L T Y Y D Y E F A Y W G Q G T L V T V S

LINKER **anti-CD3 VH**

gctggcgggggcggttccgacatcaaactgcagcagtcgggggcggagcttgctcgcccgggtgcttccgtg

A G G G G S D I K L Q Q S G A E L A R P G A S V

aaaatgtcctgtaagaccagcggctatacgttcacacgctacaccatgcattgggtgaagcagaggcctgga

K M S C K T S G Y T F T R Y T M H W V K Q R P G

cagggcctcgaatggatcggttacatcaacccctctcggggctacaccaactacaaccagaagtttaaagac

Q G L E W I G Y I N P S R G Y T N Y N Q K F K D

aaggccacgctgacaaccgacaaaagctcgtccactgcctacatgcagctgtcctcactgacctccgaagat

K A T L T T D K S S S T A Y M Q L S S L T S E D

tctgccgtgtactactgcgctcgttactacgatgaccattactgcctggactattggggccagggcacaaca

S A V Y Y C A R Y Y D D H Y C L D Y W G Q G T T

LINKER (GGS)x4 **anti-CD3VL**

```
ctgaccgtctcctccgtggagggcggcagtggaggttctggtggatccggagggtcaggtgggcgtggatgac
L  T  V  S  S  V  E  G  G  S  G  G  S  G  G  S  G  G  S  G  G  V  D  D

atccagcttacccagagtcccgctatcatgtctgcctccccaggggagaaggttactatgacctgtcgtgct
I  Q  L  T  Q  S  P  A  I  M  S  A  S  P  G  E  K  V  T  M  T  C  R  A

tccagctccgtatcttacatgaactggtaccaacagaagagcggtacttctccgaagcgttggatctatgat
S  S  S  V  S  Y  M  N  W  Y  Q  Q  K  S  G  T  S  P  K  R  W  I  Y  D

acttccaaggtggcgtccggcgtcccctatcgttttagcgggagtggctccggcaccagctactcgctgacc
T  S  K  V  A  S  G  V  P  Y  R  F  S  G  S  G  S  G  T  S  Y  S  L  T

atctcttctatggaggcggaggacgcagccacctactactgtcaacagtggtctagcaacccctgaccttc
I  S  S  M  E  A  E  D  A  A  T  Y  Y  C  Q  Q  W  S  S  N  P  L  T  F
```

HIS-Tag **Stop**

```
ggtgctggcaccaaactggagctgaagcaccaccaccaccaccattaa
G  A  G  T  K  L  E  L  K  H  H  H  H  H  H  *
```

2) Coding sequences for anti-TNC-CARs

2A) CAR-TNC5

LEADER IgK Murine **VL ST2146**

atggagaccgacaccctgctgctgtgggtgctgctgctgtgggtgccaggcagcaccggcgacatcgtgatg

M E T D T L L L W V L L L W V P G S T G D I V M

acacaagctgctcccagcgtgccagtgacacctggcgagtctgtgtccatctcttgccggagcagcaagagc

T Q A A P S V P V T P G E S V S I S C R S S K S

ctgctgcacagcaacggcaatacctacctgtactggttcctgcagaggcccggacagtctcctcagctgctg

L L H S N G N T Y L Y W F L Q R P G Q S P Q L L

atctaccggatgagcaatctggccagcggcgtgcccgatagatttctggctctggcagcggcaccgccttc

I Y R M S N L A S G V P D R F S G S G S G T A F

acactgagaatctctagagtggaagccgaggacgtgggcgtgtactactgtatgcagcacctggaatacccct

T L R I S R V E A E D V G V Y Y C M Q H L E Y P

Linker (G4S)x4

ctgaccttcggagccggcaccaagctggaactgaaaaccaaggccggaggcggaggctctggcggaggcggc

L T F G A G T K L E L K T K A G G G G S G G G G

VH ST2146

tctggcggaggcggctctggcggaggcggcagcgaaaaagtgaagctgcagcagagcggccctgagctggtt

S G G G G S G G G G S E K V K L Q Q S G P E L V

aagcctggcgcctctgtgaaggtgtcctgtaaagccagcggctacgcctttaccagctacaacatgtactgg

K P G A S V K V S C K A S G Y A F T S Y N M Y W

gtcaagcagagccacggcaagtccctggaatggatcggctacatcgacccctacaacggcgtgacctcctac

V K Q S H G K S L E W I G Y I D P Y N G V T S Y

aaccagaagttcaagggcaaagccacactgaccgtggacaagagcagctccaccgcctacatgcacctgaac

N Q K F K G K A T L T V D K S S S T A Y M H L N

agcctgaccagcgaggacagcgccgtgtactattgtgctagaggcggcggatccatctactacgccatggat

S L T S E D S A V Y Y C A R G G G S I Y Y A M D

HINGE hIgG1

tattggggccagggcaccaccgtgacagtgtctagcgatcccgccgagcccaaatctcctgacaaaactcac

Y W G Q G T T V T V S S D P A E P K S P D K T H

CH2 hIgG1

acatgcccaccgtgcccagcacctgaactcctggggggaccgtcagtcttcctcttccccccaaaacccaag

T C P P C P A P E L L G G P S V F L F P P K P K

gacaccctcatgatctcccggacccctgaggtcacatgcgtggtggtggacgtgagccacgaagaccctgag

D T L M I S R T P E V T C V V V D V S H E D P E

gtcaagttcaactggtacgtggacggcgtggaggtgcataatgccaagacaaagccgcgggaggagcagtac

V K F N W Y V D G V E V H N A K T K P R E E Q Y

aacagcacgtaccgtgtggtcagcgtcctcaccgtcctgcaccaggactggctgaatggcaaggagtacaag

N S T Y R V V S V L T V L H Q D W L N G K E Y K

CH3 hIgG1

tgcaaggtctccaacaaagccctcccagcccccatcgagaaaaccatctccaaagccaaagggcagccccga

C K V S N K A L P A P I E K T I S K A K G Q P R

gaaccacaggtgtacaccctgcccccatcccgggatgagctgaccaagaaccaggtcagcctgacctgcctg
E P Q V Y T L P P S R D E L T K N Q V S L T C L

gtcaaaggcttctatcccagcgacatcgccgtggagtgggagagcaatgggcagccggagaacaactacaag
V K G F Y P S D I A V E W E S N G Q P E N N Y K

accacgcctcccgtgctggactccgacggctccttcttcctctacagcaagctcaccgtggacaagagcagg
T T P P V L D S D G S F F L Y S K L T V D K S R

tggcagcaggggaacgtcttctcatgctccgtgatgcatgaggctctgcacaaccactacacgcagaagagc
W Q Q G N V F S C S V M H E A L H N H Y T Q K S

CD28 TM

ctctccctgtctccgggtaaaaaagatcccaaattttgggtgctggtggtggttggtggagtcctggcttgc
L S L S P G K K D P K F W V L V V V G G V L A C

CD28 cytoplasmic

tatagcttgctagtaacagtggcctttattattttctgggtgaggagtaagaggagcaggctcctgcacagt
Y S L L V T V A F I I F W V R S K R S R L L H S

gactacatgaacatgactccccgccgccccgggcccacccgcaagcattaccagccctatgccccaccacgc
D Y M N M T P R R P G P T R K H Y Q P Y A P P R

OX40

gacttcgcagcctatcgctccagggaccagaggctgccccccgatgcccacaagccccctgggggaggcagt
D F A A Y R S R D Q R L P P D A H K P P G G G S

ttccggacccccatccaagaggagcaggccgacgcccactccaccctggccaagatcagagtgaagttcagc
F R T P I Q E E Q A D A H S T L A K I R V K F S

CD3z

aggagcgcagacgcccccgcgtaccagcagggccagaaccagctctataacgagctcaatctaggacgaaga
R S A D A P A Y Q Q G Q N Q L Y N E L N L G R R

gaggagtacgatgttttggacaagagacgtggccgggaccctgagatggggggaaagccgagaaggaagaac
E E Y D V L D K R R G R D P E M G G K P R R K N

cctcaggaaggcctgtacaatgaactgcagaaagataagatggcggaggcctacagtgagattgggatgaaa
P Q E G L Y N E L Q K D K M A E A Y S E I G M K

ggcgagcgccggaggggcaaggggcacgatggcctttaccagggtctcagtacagccaccaaggacacctac
G E R R R G K G H D G L Y Q G L S T A T K D T Y

gacgcccttcacatgcaggccctgcctcctcgctaa
D A L H M Q A L P P R *

2B) ***CAR-TNC4***

CD8 Leader **VL ST2146**

atggccttaccagtgaccgccttgctcctgccgctggccttgctgctccacgccgccaggccggacgacatc

M A L P V T A L L L P L A L L L H A A R P D D I

gtgatgacacaagctgctcccagcgtgccagtgacacctggcgagtctgtgtccatctcttgccggagcagc

V M T Q A A P S V P V T P G E S V S I S C R S S

aagagcctgctgcacagcaacggcaatacctacctgtactggttcctgcagaggcccggacagtctcctcag

K S L L H S N G N T Y L Y W F L Q R P G Q S P Q

ctgctgatctaccggatgagcaatctggccagcggcgtgcccgatagatttctggctctggcagcggcacc

L L I Y R M S N L A S G V P D R F S G S G S G T

gccttcacactgagaatctctagagtggaagccgaggacgtgggcgtgtactactgtatgcagcacctggaa

A F T L R I S R V E A E D V G V Y Y C M Q H L E

Linker (G4S)x3

taccctctgaccttcggagccggcaccaagctggaactgaaaggcggcggaggaagcggaggcggaggatct

Y P L T F G A G T K L E L K G G G G S G G G G S

| **VH ST2146**

ggtggtggtggatctgaaaaagtgaagctgcagcagagcggccctgagctggttaagcctggcgcctctgtg

G G G G S E K V K L Q Q S G P E L V K P G A S V

aaggtgtcctgtaaagccagcggctacgcctttaccagctacaacatgtactgggtcaagcagagccacggc

K V S C K A S G Y A F T S Y N M Y W V K Q S H G

aagtccctggaatggatcggctacatcgaccctacaacggcgtgacctcctacaaccagaagttcaagggc

K S L E W I G Y I D P Y N G V T S Y N Q K F K G

aaagccacactgaccgtggacaagagcagctccaccgcctacatgcacctgaacagcctgaccagcgaggac

K A T L T V D K S S S T A Y M H L N S L T S E D

agcgccgtgtactattgtgctagaggcggcggatccatctactacgccatggattattggggccagggcacc

S A V Y Y C A R G G G S I Y Y A M D Y W G Q G T

CD8 Stalk

accgtgacagtgtctagcaccacaacacccgctcctagacctccaacaccagctccaacaatcgccagccag

T V T V S S T T T P A P R P P T P A P T I A S Q

cctctgtctctgaggccagaagcttgtagacctgctgctggcggagccgtgcatacaagaggactggacttc

P L S L R P E A C R P A A G G A V H T R G L D F

CD8 TM

gcctgcgacatctacatctgggctcctctggctggcacatgcggagtgttgctgctgagcctggtcatcacc

A C D I Y I W A P L A G T C G V L L L S L V I T

CD8cyt **4-1BB**

ctgtactgcaagcggggcagaaagaaactgctctacatcttcaagcagcccttcatgcggcccgtgcagacc

L Y C K R G R K K L L Y I F K Q P F M R P V Q T

CD3z

acacaagaggaagatggctgctcctgcagattccccgaggaagaagaaggcggctgcgagctgagagtgaag

T Q E E D G C S C R F P E E E E G G C E L R V K

ttcagcagatccgccgacgctcctgcctatcagcagggacagaaccagctgtacaacgagctgaacctgggg

```
F  S  R  S  A  D  A  P  A  Y  Q  Q  G  Q  N  Q  L  Y  N  E  L  N  L  G

agaagagaagagtacgacgtgctggacaagcggagaggcagagatcctgagatgggcggcaagcccagacgg
R  R  E  E  Y  D  V  L  D  K  R  R  G  R  D  P  E  M  G  G  K  P  R  R

aagaatcctcaagagggcctgtataatgagctgcagaaagacaagatggccgaggcctacagcgagatcgga
K  N  P  Q  E  G  L  Y  N  E  L  Q  K  D  K  M  A  E  A  Y  S  E  I  G

atgaagggcgagcgcagaagaggcaagggacacgatggactgtaccagggcctgagcaccgccaccaaggat
M  K  G  E  R  R  R  G  K  G  H  D  G  L  Y  Q  G  L  S  T  A  T  K  D

acctatgatgccctgcacatgcaggccctgcctccaagataa
T  Y  D  A  L  H  M  Q  A  L  P  P  R  *
```

3) *Sequences of furin-T2A linkers*

3A: in *EGFRxCD3/TNC5*

```
Furin site  linker   T2A
cgccgcaagcgcggcagcggtgagggccgtgggtctctgctaacctgcggggatgtagaggagaacccgggc
R  R  K  R  G  S  G  E  G  R  G  S  L  L  T  C  G  D  V  E  E  N  P  G

cct
P
```

3B: in *TNC5/EGFRxCD3*

```
Furin site  linker   T2A
cggcggaagcgtggttcgggcgagggccggggctcgctgctgacctgcggggacgtggaggagaatcccggg
R  R  K  R  G  S  G  E  G  R  G  S  L  L  T  C  G  D  V  E  E  N  P  G

cca
P
```

4) *Coding sequence of TNC-TM*

```
HB-EGF Signal Peptide
atgaagctgctgccatctgtggtgctgaagctgtttctggccgccgtgctgtctgcactggttacaggcgag
M  K  L  L  P  S  V  V  L  K  L  F  L  A  A  V  L  S  A  L  V  T  G  E
HB-EGF N-Terminal
agcctggaacggctgagaagaggacttgccgccggaaccagcaatcccgatcctcctaccgtgtccaccgat
S  L  E  R  L  R  R  G  L  A  A  G  T  S  N  P  D  P  P  T  V  S  T  D

cagttgctgcctcttggcggcggacgggatagaaaagtgcgggatctgcaagaggccgacctggacctgctg
Q  L  L  P  L  G  G  G  R  D  R  K  V  R  D  L  Q  E  A  D  L  D  L  L

agagtgacactgtctagcaagccccaggctctggccacacctaacaaagaggaacacggcaagcgcaagaag
R  V  T  L  S  S  K  P  Q  A  L  A  T  P  N  K  E  E  H  G  K  R  K  K
                     TNC
aaaggcaaaggcctggggaagtgcgtgtgcgagcctggatggaagggccctaattgcagcgagcctgagtgc
K  G  K  G  L  G  K  C  V  C  E  P  G  W  K  G  P  N  C  S  E  P  E  C
```

cctggcaactgtcacctgagaggcagatgcatcgacggccagtgcatctgcgacgatggattcacaggcgag
P G N C H L R G R C I D G Q C I C D D G F T G E

gactgctcccagctggcctgtcctagcgactgcaacgatcagggcaaatgcgtgaacggcgtgtgcatctgt
D C S Q L A C P S D C N D Q G K C V N G V C I C

ttcgagggatacgctggcgccgactgcagcagagaaatctgccctgtgccatgcagcgaggaacatggcaca
F E G Y A G A D C S R E I C P V P C S E E H G T

tgtgtggatggcctgtgcgtgtgtcacgatggctttgctggcgacgattgcaacaagcccctgtgcctgaac
C V D G L C V C H D G F A G D D C N K P L C L N

aactgctacaaccggggcagatgcgtggaaaacgagtgtgtgtgcgacgagggctttaccggggaagattgc
N C Y N R G R C V E N E C V C D E G F T G E D C

tccgagctgatctgccccaacgactgcttcgacagaggccggtgcatcaacggcacctgttactgcgaggaa
S E L I C P N D C F D R G R C I N G T C Y C E E

ggcttcactggcgaagattgtggcaagcccacatgtccccacgcctgtcacacacaaggcagatgtgaagag
G F T G E D C G K P T C P H A C H T Q G R C E E

ggccagtgtgtctgtgatgagggatttgccggcgtggactgtagcgagaagagatgccctgccgactgccac
G Q C V C D E G F A G V D C S E K R C P A D C H

aatagaggacgctgtgtggacggaagatgcgagtgtgacgacgggtttacaggcgccgattgtggcgagctg
N R G R C V D G R C E C D D G F T G A D C G E L

aagtgcccaaatggctgttctggccatggaagatgtgtgaacggacagtgcgtttgtgatgaaggctacacc
K C P N G C S G H G R C V N G Q C V C D E G Y T

ggcgaggattgcagccagctgagatgtcccaacgattgccacagccgtgggagatgtgtggaaggcaaatgt
G E DC S Q L R C P N D C H S R G R C V E G K C

gtctgcgagcagggcttcaagggctacgactgctccgatatgagctgccctaatgattgccaccagcacggt
V C E Q G F K G Y D C S D M S C P N D C H Q H G

cgctgtgtgaatgggatgtgcgtttgcgacgacggatacacaggggaagattgccgggacagacagtgcccc
R C V N G M C V C D D G Y T G E D C R D R Q C P

agagactgtagcaataggggcctgtgtgtcgatgggcagtgtgtttgcgaagatgggttcaccggacctgac
R D C S N R G L C V D G Q C V C E D G F T G P D

tgtgccgagctgtcctgtccaaatgactgccacggacaggggagatgcgtcaacggtcaatgcgtgtgccat
C A E L S C P N D C H G Q G R C V N G Q C V C H

gagggcttcatgggaaaagactgcaaagaacagcggtgcccctccgactgtcatggccagggacgttgtgtt
E G F M G K D C K E Q R C P S D C H G Q G R C V

gatggccaatgtatctgccacgaggggttcactggcctggattgtggccagcacagctgcccaagcgactgt
D G Q C I C H E G F T G L D C G Q H S C P S D C

aacaacctgggacagtgtgtgtccggccgctgcatctgtaatgagggctatagcggagaggactgttccggc
N N L G Q C V S G R C I C N E G Y S G E D C S G

HB-EGF like domain **HB-EGF TM**

ctgtctggccctgtggaaaaccggctgtacacctacgaccacaccaccatcctggctgtggtggcagtggtg
L S G P V E N R L Y T Y D H T T I L A V V A V V

HB-EGF Cytoplasmatic

ctgtctagcgtgtgtctgctggttatcgtgggcctgctgatgttcagataccatcggagaggcggctacgac
L S S V C L L V I V G L L M F R Y H R R G G Y D

gtggaaaatgaggaaaaggtggccctgggctga
V E N E E K V A L G *

5) ***Primer sequences used for qRT-PCR of human TNC mRNA***

a) Forward primer

5′ – ACTGTGGACGGAACCAAGAC – 3′

b) Reverse Primer

5′ – TGATGTTGGCTGTCACCAGG – 3′
